# Quantifying COVID-19 policy impacts on subjective well-being during the early phase of the pandemic: A cross-sectional analysis of United States survey data from March to August 2020

**DOI:** 10.1371/journal.pone.0291494

**Published:** 2023-09-21

**Authors:** Ke Shen, Mayank Kejriwal

**Affiliations:** Department of Industrial and Systems Engineering, University of Southern California, Los Angeles, California, United States of America; Universite Paris Pantheon-Assas, FRANCE

## Abstract

To stop the spread of COVID-19, a number of public health policies and restrictions were implemented during the pre-vaccination phase of the pandemic. This study provides a quantitative assessment of how these policies impacted subjective well-being (SWB) in the United States over a 6-month period spanning March to August 2020. We study two specific research objectives. First, we aim to quantify the impacts of COVID-19 public health policies at different levels of stringency on SWB. Second, we train and implement a conditional inference tree model for predicting individual SWB based both on socio-demographic characteristics and policies then in place. Our results indicate that policies such as enforcing strict stay-at-home requirements and closing workplaces were negatively associated with SWB, and that an individual’s socio-demographic characteristics, including income status, job, and gender, conditionally interact with policies such as workplace closure in a predictive model of SWB. Therefore, although such policies may have positive health implications, they also have secondary environmental and social implications that need to be taken into account in any cost-benefit analysis of such policies for future pandemic preparedness. Our proposed methodology suggests a way to quantify such impacts through the lens of SWB, and to further advance the science of pandemic preparedness from a public health perspective.

## Introduction

While vaccination and better understanding of SARS-CoV-2 (COVID-19) has helped resume everyday life in the United States and the rest of the world [1, 2], the early phase of the pandemic (especially, the first three quarters of 2020) was marked by a general sense of fear and uncertainty. COVID-19 hospitalizations and deaths rose rapidly in many parts of the world, and the economic toll on entire segments of the population was also devastating [3, 4]. In response, both local and national governments devised a range of policy measures to contain the virus. While some policies may have been highly local, within the United States, common policies (often implemented at the state level, at different ‘stringency’ levels) included workplace and school closures, as well as restrictions on gatherings.

While originally devised as beneficial health interventions, such policy measures can also have broad indirect effects on public sentiment and subjective well-being. This was most recently evidenced in the widespread protests that broke out in China in late 2022, and the subsequent backtracking by the government on strict policies of lockdown. Beyond China, there has been a general recognition that not all policies were equally effective, and that some policies may have had deleterious effects on public mental health [5, 6], even as they led to lower COVID-19 infection rates.

In both social science and psychology, subjective well-being (SWB) has become an important topic of research in the last few decades [7–10]. Diener defines SWB as an “an umbrella term used to describe the level of well-being people experience according to their subjective evaluations of their lives.” Such evaluations can be positive or negative, and include judgments about life satisfaction, as well as affective reactions such as joy, worry and enjoyment. Although not equivalent to mental health, SWB has been widely used to gauge the public’s ‘happiness’ levels across time and space [11]. However, studies on SWB, and their relationship to COVID-19 policies, remain relatively sparse, although they are growing [12, 13]. For example, Gehle et al. investigate well-being among older adults in Germany during the pandemic [14]. Another recent study includes a longitudinal analysis of ‘happiness’ data during COVID-19 in Italy [15], and a panel-based study of the effects of the pandemic on livelihoods in Côte d’Ivoire [16]. Specific aspects of SWB (e.g., work-life balance satisfaction), both cultural and economic, have also been studied [17, 18]. Drivers of SWB, such as physical activity and financial satisfaction [19], have also begun to be studied in greater detail in specific regional contexts and populations (e.g., adolescents in Canada [20]).

As the studies above demonstrate, the related literature on this subject, in the context of COVID-19, has tended to focus on specific regions and settings, and on variables that are related to policy impacts but that do not directly encode the stringency at which a policy was implemented. While investigating the ‘first-order’ health and economic impacts of various COVID-19 policies is clearly important [21–24], it is also important to measure the effect on public SWB of containment policies in a larger country where the policies were implemented in a heterogeneous fashion (i.e., at different stringency levels).

This article directly investigates the association between the stringency levels of pandemic-era policies, and SWB, across the United States. We design our study with two objectives in mind. Both objectives are designed to contribute to the growing literature on effective pandemic preparedness that takes a more comprehensive view than rather than just measuring health outcomes of pandemic containment policies. The first objective is to quantify the effects of different policies (such as strict stay-at-home requirements), implemented at different stringency levels in different states, on SWB. Because socio-demographic variables, such as gender and employment status, are also known to be important drivers of SWB [25], we conduct our analysis by attempting to control for these other variables. We use both partial correlations and fixed-effects regressions for this purpose. In the fixed-effects regression, the key independent variable is the stringency level of a single policy, with the socio-demographic covariates and state serving as control variables. The dependent variable is always the SWB. By conducting such regressions, one for each policy, we are able to get deeper insights into their individual impacts on SWB, even when other covariates are included in the model.

In contrast with the first objective, which is more explanatory, the second objective seeks to predict an individual’s SWB using not only the individual’s socio-demographic features, but also the policies in place in the location (state) where the individual is residing. We do so by building a conditional inference tree model that is able to take complex statistical dependencies between the different variables into account, and only includes relationships in the tree that are statistically significant above a minimum level. More details on this model will be provided subsequently. The use of conditional inference trees in computational social science and public health is relatively novel, but has some precedent, both in the vaccine hesitancy and in the climate skepticism literature [26, 27]. In investigating the second objective, we show that its utility can be further extended to better predict individual response (through a measure like SWB) to public restriction policies.

Although both the conditional inference tree model, and more classic methods like the fixed-effects regression, cannot be used in this article to make strong causal claims, they can be used to serve a limited explanatory (first objective), and a predictive (second objective), goal. One reason that a causal model has proven difficult to build in the COVID-19 policy literature, is that the the data collected during the pandemic was largely observational, and by necessity, did not follow protocols (such as randomization and blinding) designed to support rigorous causal inference. Nevertheless, through a careful study of the results yielded by rigorous and established statistical models, and a discussion of their relation to similar findings from other parts of the world, this article aims to advance the science of pandemic preparedness and subsequently, inform government policy when considering similar such restrictions in the future.

## Methods

### Ethics

The study, with IRB number of UP-21–00692, was submitted on 8/10/2021 and received written approval by the University of Southern California Institutional Review Board on 9/29/2021. We attach the submitted protocol, the approval notice, and the Gallup COVID-19 survey methodology documentation. Note that the study is retrospective, and utilized data already collected by Gallup, which obtained consent from all participants whose data was used for this research. The IRB provided us with an ethical waiver for the study.

### Sample and data

This study is based on the joint analysis of two data sources: survey data collected by Gallup, and policy-response data compiled by the University of Oxford. Each of these is described in turn below. As the study is an observational cross-sectional analysis, the STROBE checklist is included as S1 Table in S1 File.

#### Data source 1 (Gallup-panel COVID-19 survey)

Gallup began fielding on March 13, 2020 by launching a specific survey that collected people’s responses during the COVID-19 pandemic, polling daily random samples of the Gallup Panel (a probability-based, nationally representative panel of U.S. adults). As in other Gallup surveys (such as the Gallup World Poll [28]), questions measured aspects of subjective well-being (SWB), as well as other socio-demographic details such as employment status, job area, and political affiliation.

In the literature, SWB has been measured in a variety of ways, but two that have extensively used are *life satisfaction* [29, 30] and *affective well-being* [31, 32]. For our study, we obtained individual-level data points for both these measures from Gallup. The inclusion of both measures allows us to address our research questions comprehensively and verify the robustness of our research findings. While the main text primarily focuses on reporting the results related to life satisfaction, we provide the results concerning affective well-being in the supplementary materials. In this way, we can ensure that our study captures a comprehensive understanding of SWB from multiple dimensions.

To measure life satisfaction, the following question was asked in the Gallup survey (marked as the WP16 question in the survey): *Please imagine a ladder with steps numbered from* ***zero*** *at the bottom to* ***ten*** *at the top. The top of the ladder represents the best possible life for you and the bottom of the ladder represents the worst possible life for you. On which step of the ladder would you say you personally feel you stand at this time?* The response to this question is always recorded on a eleven-point scale, from the worst possible (0) to the best possible (10). We henceforth refer to this variable as *SWB-ladder*, the question above as the *life satisfaction* question.

From March 13, 2020 to August 30, 2020, Gallup polled 9,285 responses for each of these SWB-related questions from their representative sample of US adults. During the period, we observed a noteworthy decrease of 0.89 in the mean life satisfaction reported by respondents, based on the collected data points. Descriptive analysis shows that 58.2 percent of respondents were male and 82 percent of participants were employed full-time. About 68 percent of participants were in a household that earned $48,000–180,000 per year. 70.8 percent of the respondents were aged below 60. Democrats were 54.1 percent of participants, while Republicans were 45.9 percent.

#### Rationale for case selection

The Gallup data used for this study constitutes a representative sample of US adults. We chose to focus on the US both because of data availability, and Gallup’s rigorous methodology for assigning weights to samples, but also to control for any country-based differences (including, but not limited to, cultural and legal differences) that might serve as explanations for our findings. Because cases are from only a single country, location-based differences can only arise due to (for example) the state in which a respondent is based, and can be controlled for using fixed effects. It also allows us to more rigorously control for socio-demographic features such as income and political affiliation as levels within these variables (e.g., ‘Republican’ or ‘high income’) *mean* the same thing, at least to a first approximation, for respondents surveyed within a single nation with a federalist structure. Methodologically, any results obtained have (arguably) less chance of unknown confounding or bias, and are likely to be robust. Our work has precedent in other studies that have also been applied in specific regional contexts (as subsequently covered in Discussion).

Additionally, the case selection is restricted to the period from March 2020 to August 2020 for several important reasons. First, compliance with policies was generally high in the early days of the pandemic due to the uncertainty and fear associated with its impacts, and the high infection and death counts being reported all over the world. Therefore, an association between SWB and policy stringency, if any, is likely to be most reliable in this period, allowing us to get a more accurate estimate of the strength of this association than in later periods. Second, because vaccines against COVID-19 had not been invented and rolled out to the public yet, containment policies were the only measures that public health agencies could adopt to slow the spread of the virus. Later studies showed that vaccination, as well as vaccine hesitancy, were associated with SWB [27, 33], and this can confound the association between policy stringency and SWB. Last, but not least, within the United States, the period after November 2020 was one of political tumult, leading to incidents such as the January 6 Capitol Riots, and subsequent hearings and indictments on the matter. As the Democrat and Republican parties have different views on COVID-19 policy implementation, the confounding effect of political affiliation is likely to strengthen post-2020 presidential election. In a Brazilian study [34], the impact of partisan political divide was also emphasized. Due to all of these reasons, we determined that restricting analysis to the window of time between March and August 2020 was best suited for investigating the research objectives stated earlier.

#### Data source 2 (Oxford COVID-19 Government Response Tracker)

The Oxford Covid-19 Government Response Tracker (OxCGRT) collects systematic data on well-defined policy measures that governments have taken in response to COVID-19, and when they have taken those measures [35]. Specifically,it reports the extent of government action on each policy using a *stringency scale* with lower values (e.g., 0) on the scale indicating no action, and progressively higher values indicating more stringent action. Table 1 provides the description and acronyms of the full set of COVID-19-related policies studied in this work. The stringency levels of these policy measures recorded in OxCGRT are provided in S2 Table in S1 File.

**Table 1:** Description and acronyms of COVID-19-related policies recorded in the OxCGRT dataset and studied in this work.

Acronym	Policy	Description
SC	School closing	Require school and university closures
WC	Workplace closing	Require workplace closures
CE	Cancel public events	Require cancelling public events
GR	Restrictions on gatherings	Limitation on gatherings
TC	Close public transport	Require the closing of public transport
SHR	Stay at home requirements	Orders to “shelter-in-place” and otherwise confined to the home
IMR	Restrictions on internal movement	Restrictions on internal movement between cities/regions
TP	Testing policy	Policy on who has access to testing
CT	Contact tracing	Policy on contact tracing after a positive diagnosiss

OxCGRT is daily updated for each of the policy measures that it tracks at the state level in the US. It also includes data on confirmed cases, deaths, hospitalizations, testing, as well as other pandemic-related variables of potential interest.

#### Data concatenation

Finally, we describe the procedure for ‘concatenating’ or joining these two datasets to obtain a more comprehensive dataset in service of the two research objectives. We note first that Gallup used landline, cellphones, and address-based sampling methods to randomly collect data from its participants. The obtained samples are weighted to match the national demographics of gender, age, region, and so on. Information such as the state that respondents lived in, and the timestamp when the survey ended, are also included to ensure that the samples are properly representative of adults in the United States. To track changes in policy measures, OxCGRT also continuously records timestamps and state information in the data. Hence, these two data sources can be concatenated or ‘joined’ using the timestamps and states as joining keys. Following such concatenation, we obtain stringency levels of different COVID-19 policy measures that applied at different times during our study for individuals living in the corresponding states. In aggregate, the joined data can be used to quantify the effects of COVID-19 policies on individual SWB while controlling for a range of variables. The minimal data set for replicating the results of this study may be found in [36]. Other methodological steps (including data pre-processing) for obtaining the key measures of variables used in the study are described in the next section.

### Measures of variables

The concatenated dataset contains data for both socio-demographic and SWB variables at the individual level, along with aggregate (i.e., state-level) timestamped information on the confirmed COVID-19 cases and deaths per day. Concerning the latter, the SWB-ladder question is technically a categorical variable that records values from 0 to 10. Because this is an ordinal and relatively fine-grained numerical scale, similar to earlier work on SWB [37], we treat it as real-valued in our analysis rather than categorical.

Regarding the life satisfaction reported by respondents in the collected samples, the mean life satisfaction is 6.89, with a standard deviation of 1.57. The SWB-ladder variable exhibits a negative skewness of -0.75, indicating a left-skewed distribution with a longer tail on the left side. The positive kurtosis coefficient (0.69) suggests that the SWB-ladder distribution has slightly heavier tails and a sharper peak compared to a normal distribution. S3 Table in S1 File includes summary statistics for the responses to the life satisfaction and other SWB-related variables (affective well-being questions). In the remainder of this study, we only consider the SWB-ladder variable as our measure of SWB; however, where relevant, we also provide results in the supplementary material for measures like *Enjoyment* and *Worry* to support the robustness of the primary result.

Among the 12 socio-demographic and COVID-based variables that we consider, four are real-valued (age, number of children in household, confirmed COVID-19 cases per day, and COVID-19 deaths per day) and eight are categorical. The mean age of the respondents is 51.65 years, with the youngest respondent being 20 years old and the oldest being 105 years old. The standard deviation of 12.08 indicates relatively moderate dispersion around the mean age. The mean number of children in the household among respondents is 0.68, ranging from 0 to 10 children. The positive skewness of 1.89 indicates a rightward tail, implying a relatively higher number of households with fewer children. Summary statistics of socio-demographic and COVID-based variables and their levels in the pre-processed dataset are provided in S4 Table in S1 File.

### Models and data analysis

In this section, we describe the models and data analysis procedures that were implemented to empirically investigate both objectives. Before describing these procedures, we note that, in some cases, the categories described in the previous section were found to be too fine-grained for statistically robust studies. Specifically, income and job areas serve as examples of such finely-divided categories. For example, the original response to the income variable is on a 1 to 10 scale, where 1 stands for *less than $12,000*, 2 stands for *$12,000 to $23,999*, all the way to 10, which stands for *$240,000 and over*. Based on the statistics reported by the Pew Research Center [38], and their income designation, we collapse these ten levels of the variable into three coarser-grained levels: household incomes that are less than $47,999 are placed in the category of *low income*, incomes between $48,000 and $179,999 are classed as *middle income*, and incomes that are equal to or higher than $180,000 as *high income*.

Similarly, we applied a similar procedure to the job areas, which initially had 20 categories. Using analysis from the U.S. Bureau of Labor Statistics [39] and other relevant reports [40, 41], we collapsed these fine-grained categories into three levels: *low-affected, middle-affected*, and *high-affected* jobs. Job areas such as hospitality, transportation, and retail are categorized as highly affected by the pandemic, while jobs in government, public policy, and finance are classified as moderately affected by COVID-19. Lastly, agriculture, law, and other job areas are considered as low-affected jobs. Further details about the categorized job areas can be found in the S5 Table in S1 File.

Next, we discuss the specific models used in the study.

#### Estimating impact of policy stringency on SWB

Using the concatenated dataset, we index observations by date *t* and individual *i* in order to distinguish between the observations of an individual *i* at various points in time. In other words, there can be multiple records for individual *i* in the dataset, but there is at most one record given both *t* and *i* (which together serve as the primary key of the dataset). This is necessary because the sampling methodology of Gallup is structured such that the same respondent can appear in the dataset multiple times, at different points in time. Within the Gallup codebook, the panel data is denoted as the “unbalanced panel data set”, although the codebook concedes that the imbalance does not typically have a significant impact on analyses using this data [42].

To get an initial overview of the effect of each COVID-19 policy, we compute a partial correlation (using the Spearman’s rank correlation measure) between each SWB variable and each COVID-19 policy measure. The correlation is partial because in each of our results, we control for (and explicitly specify) a common socio-demographic variable, such as income. The Spearman’s rank correlation is a non-parametric measure of statistical dependence between the rankings of two variables. It ranges from -1 to 1 and assesses how well the relationship between two variables can be described using a monotonic function. If the correlation between an SWB variable (say *SWB-ladder*) and a policy measure is close to 1, it implies that people would experience higher life satisfaction if this policy measure were to be implemented more stringently.

As noted earlier, when reporting results, we control for some socio-demographic variables to evaluate whether significant differences in association (between SWB and policy measure) are observed among different segments of the population. More precisely, we use three socio-demographic variables (income, party affiliation, and job area) as controls. Each of these is categorical: income is divided into three levels (low-income, middle-income, and high-income) using the methodology described earlier, party affiliation into two levels (Democrat and Republican), and job area into three levels (low-affected, middle-affected, and high-affected).

As mentioned earlier, we only report the associations between each policy measure and the *SWB-ladder* variable in the main text. However, we also test for robustness by reproducing similar (partial correlation) results for related SWB variables such as *Enjoyment* and *Worry* in the S6–S8 Tables in S1 File.

To further study the impact of COVID-19 policy measures, we also conduct a series of fixed-effects regressions with *SWB-ladder* as the dependent variable and the stringency level of a specific policy (with level 1 to *l*, where *l* depends on the policy) as the independent variable. Control variables for the regression include socio-demographic variables, as well as the two COVID-based variables (confirmed cases and deaths), and with dummy variables representing states. The equation may be given as:

$$\begin{array}{cc} SWB_{i,t}= & \beta_{0}+\beta_{1}age_{i}^{2}+\beta_{2}child_{i}+\beta_{3}confirmed_{t,state_{i}}+\beta_{4}death_{t,state_{i}}+\\ & \alpha_{1}male_{i}+\alpha_{2}part\_time_{i}+\alpha_{3}has\_young\_child_{i}+\alpha_{4}has\_old\_child_{i}+\\ & \alpha_{5}middle\_income_{i}+\alpha_{6}high\_income_{i}+\alpha_{7}republic_{i}+\\ & \alpha_{8}job\_1_{i}+\cdots +\alpha_{26}job\_19_{i}+\alpha_{27}state\_1_{i}+\cdots +\alpha_{76}state\_50_{i}+\\ & \alpha_{77}policy\_L{\left(1\right)}_{t,state_{i}}+\cdots +\alpha_{77+l}policy\_L{\left(l+1\right)}_{t,state_{i}}+\delta_{t}+u_{it} \end{array}$$ (1)

Here, *β*_0_ represents the regression intercept, which is an estimate of the *reference SWB* given zero (or default) values for all of the explanatory variables. To interpret the intercept, we note that, for categorical variables such as gender (with possible values *male* and *female*) and employment situation (with possible values *part-time* and *full-time*), we only specify a dummy variable for a single category (say, *male* and *part-time*, respectively) in the data set. Dummy variables for *female* and *full-time* are excluded to avoid collinearity. If gender and employment situation were the only explanatory variables, and we set *male*_*i*_ = 0 and *part*_*time*_*i*_ = 0, *β*_0_ becomes the estimated average SWB of women with full-time jobs. For the other (categorical) variables in the regression equation above, the first level shown in the *Level* column in S4 Table in S1 File (e.g., *female* for the *gender* variable) is always the reference.

In the equation above, *β*_*j*_(*j* ≠ 0) represents the estimated effect of a continuous numeric variable (such as *age*) on an individual’s SWB, for fixed values of the other variables in the equation. Analogously, *α*_*j*_ represents the estimated effect of a dummy variable, and represents a *fixed effect* as it is independent of the date. Recall that, because our research question is to study the impact of COVID-19 policies on individuals’ SWB, we conduct a detailed analysis on the each of the fixed-effects variables *α*_*j*_, with *j* ranging from {77, …, 77 + *l*}, with *l* varying by policy. The index *j* begins from 77 because there are 76 non-fixed effects variables (26 socio-demographic variables, and 50 state variables).

Finally, *δ*_*t*_ estimates the average change in the SWB, keeping all other variables fixed, on date *t* relative to March 13, 2020 (which is the reference). We denote *δ*_*t*_ as a *date fixed-effect* variable because it is independent (at a fixed date) of the participant index *i*: in other words, the ‘effect’ of date *t* is ‘fixed’ across all individuals.

#### Conditional inference tree model for predicting individual SWB

To quantify conditional effects between the socio-demographic variables and policy measures on SWB, we implement a *classification and regression tree* (CART) model called the conditional inference tree (CIT) on the concatenated dataset. The *traditional* CART model is an implementation of recursive partitioning, which has been applied across fields ranging from disease diagnosis [43, 44] to predicting the spread of infectious diseases [45, 46], and exploring the social determinants of mental health [47]. Tree-structured models have the advantage that their results can be used to analyze and interpret intuitively the interactive effects of independent variables on a target variable (such as SWB-ladder).

However, the traditional CART model is susceptible to overfitting and selection biases, as it has been found to favor covariates with more potential splits [48]. CIT models resolve these issues by applying suitable statistical tests to variable selection strategies and split-stopping criteria [49]. The CIT method has been employed in various contexts, such as environmental science [50–52], and evaluating treatment strategies associated with diseases [53, 54].

To train the CIT model for our study, we randomly shuffle half the data points (4,643) from the concatenated dataset. The remaining 4,642 data points are treated as a test set used to evaluate the trained model. Based on the tree structure, we can then analyze the conditional and interactive effects of various policy measures on individuals’ SWB-ladder assessment, while considering also the influence of socio-demographic characteristics, such as marital and income status.

## Results

To study the relative influence of each COVID-19 policy measure on an individual’s SWB, we first analyzed the partial correlation between the stringency level of each policy measure and *SWB-ladder* in different socio-demographic groups. The partial correlation results between policy stringency and two other affective well-being variables (*Enjoyment* and *Worry*) are included in S9 and S10 Figs in S1 File.

**Fig 1:**
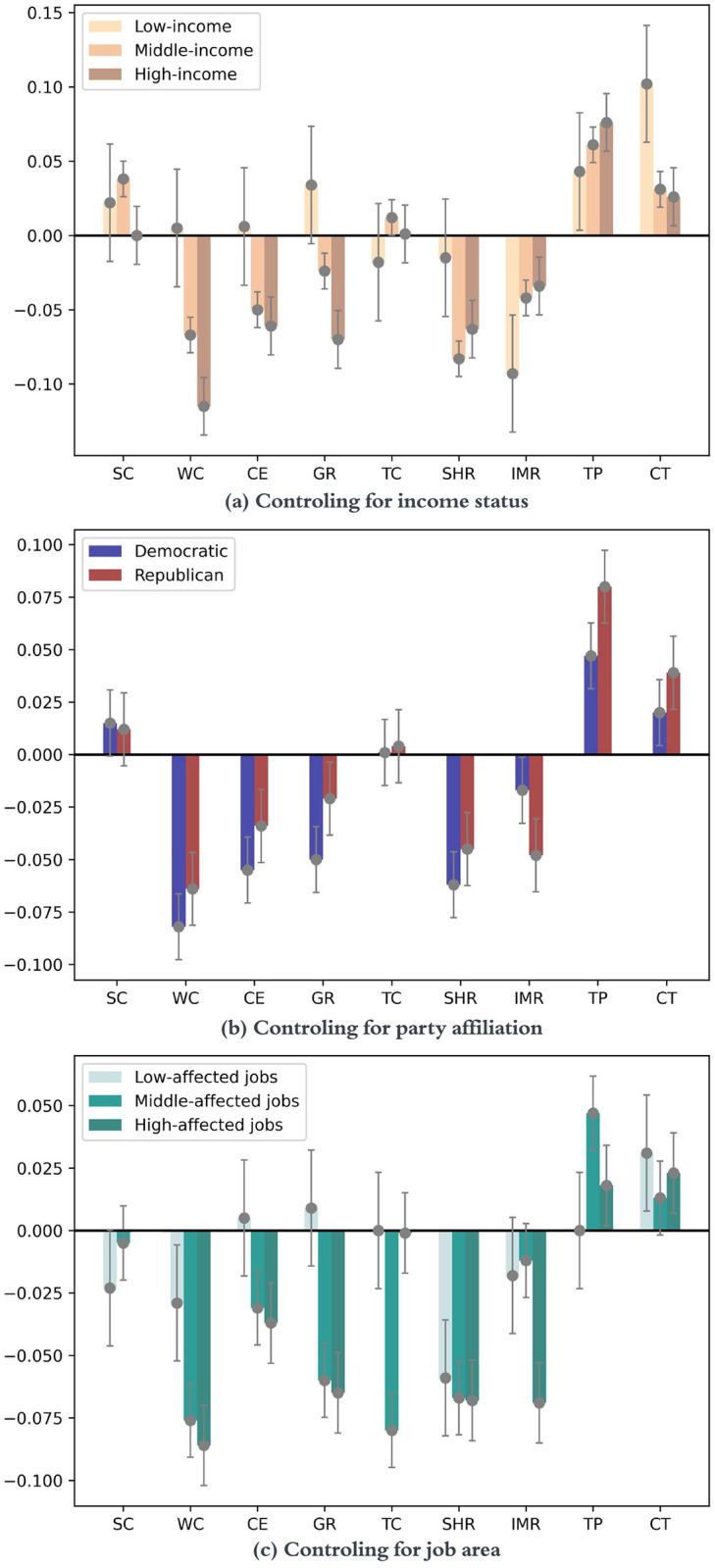
The partial correlation (y-axis) between SWB-ladder variable and each COVID-19 policy measure (x-axis) after controlling for respondent’s income status (a), party affiliation (b), and job areas (c). No correlation was observed between the school closing policy and the life satisfaction of respondents with job areas highly impacted by COVID-19 as the policy remained constant at level 3 across all corresponding data points. Error bars represent the standard errors of the correlation coefficients. Policy acronyms were enumerated earlier in Table 1.

Fig 1 illustrate the partial correlation when controlling for income, party affiliation and job areas, respectively. For the income, the respondents are divided into three groups based on annual household income: low-income, middle-income, and high-income. The results show that strict *stay at home requirements* (SHR) and *internal movement restrictions* (IMR) are negatively correlated with people’s life satisfaction, with average correlation of -0.054 (*P* < 0.01) and -0.056 (*P* < 0.10) with the *SWB-Ladder* variable, respectively. The converse is observed with the *contact tracing* (CT) policy. All else being equal, a stringent CT policy is associated with higher life satisfaction, with average correlation 0.053 (*P* < 0.05) across all groups.

The figure also suggests that the low-income group tends to have different SWB responses (compared to high-income and middle-income groups) when policies change. For example, when the *workplace closure* (WC) policy is adjusted to a more stringent level, such as from requiring certain departments to close all but essential workplaces, life satisfaction shows a negative association for middle-income and high-income group, while the association is opposite for the low-income group.

Concerning the partial correlations controlling for respondents’ party affiliation, initially, we expected different trends in life satisfaction between the two groups when adjusting the stringency level of some COVID-19 policies. However, the results show that the direction of the association is typically congruent between the two groups, but the magnitude of the correlation can still differ quite a bit. Among Democrats, the association between *SWB-ladder* and the stringency of the CT policy is 0.02 (*P* = 0.17), but for Republicans, the association is much higher 0.039 (*P* < 0.05). Interestingly, policies such as *testing policy* (TP) and *school closure* (SC) are still observed to have positive effects on people’s SWB, consistent with the partial correlation results when controlling for income status. One important point that must be borne in mind when interpreting some of these results is that they only apply to the time-period in which the data was collected, which was still the early period in the crisis and prior to a vaccine being developed.

When we divided respondents by their job areas, an obvious trend can be seen with the WC and SHR policy measures: more stringent policies are more negatively (and highly significantly) correlated with life satisfaction for individuals employed in job areas that were moderately to highly affected by COVID-19, such as people working in *College or University*, *Hospitality* or *Retail*. However, the TP policy showed a positive association with the life satisfaction measure for those working in job areas moderately impacted by the pandemic or more severely affected. Not surprisingly, in most cases (except SC, IMR, and TP policies), individuals engaged in low-affected jobs, such as agriculture, insurance, and law, tend to experience the most positive effects or the least negative impact in their life satisfaction when the corresponding policy is adjusted to be more stringent.

The correlation distribution specific to each job area is illustrated in heatmaps provided in the S11–S13 Figs in S1 File. Upon closer examination of the correlation between changes in COVID-19 policy and the life satisfaction of individuals within each distinct job area, we find that the stringent WC policy is associated with greater worry, especially for those working in *Construction*, *Retail, Utilities*, and *Training or Library*. The average association across the four groups is 0.156 (*P* < 0.01). Clear evidence suggests that, with further tightening of COVID-19 policies, notably WC, GR, and SHR, respondents engaged in *Arts, Sports and Media* are unable to enjoy life as they did before the pandemic. The Spearman’s correlation between the GR measure and the life satisfaction of artists, athletes, and media practitioners is as low as -0.196 (*P* < 0.01).

As expected, *Retail* is one of the most vulnerable industries affected by the pandemic. The stringent WC and IMR policies dramatically reduce the life satisfaction of people engaged in retail (with correlation -0.197 and -0.199, respectively; *P* = 0.17). However, the stringent TP and CT polices, as mentioned before, tend to be positively associated with SWB (with correlation 0.207 and 0.140, respectively; *P* < 0.10).

### Objective 1 (modeling policy impacts on SWB with fixed-effects regression)

Tables 2 and 3 report the results of the fixed-effects regression models for *SWB-ladder*, controlling for variables such as COVID-19 policy measures and socio-demographic variables.

**Table 2:** Fixed-effects regression coefficients of three COVID-19 related policies: School closing (SC), workplace closing (WC), and cancel events (CE), and socio-demographic variables.

			SC	WC	CE
Dummy Variables	Policy (Compared to Level 0)	Level 2	-	0.077	0.094
		Level 3	-1.229	0.033	-
	Gender (Compared to Female)	Male	0.210 ***	0.211 ***	0.209 ***
	Employed Situation (Compared to Full-time)	Part-time	-0.084	-0.082	-0.084
	Job Area (Compared to Low-affected Jobs)	Middle-affected	0.156 **	0.156 **	0.156 **
		High-affected	0.095	0.093	0.093
	Has Young Children (Compared to No)	Yes	-0.173 **	-0.173 **	-0.174 **
	Has Older Children (Compared to No)	Yes	-0.186 **	-0.188 **	-0.186 **
	Income (Compared to Low-income)	Middle-income	0.560 ***	0.558 ***	0.558 ***
		High-income	0.885 ***	0.883 ***	0.882 ***
	Party (Compared to Democrat)	Republican	0.287 ***	0.285 ***	0.285 ***
	State (Compared to AK)	NH	-0.191	-0.233	-0.188
		MI	1.234	1.178	1.284
Numeric Variables	Age		0.010 ***	0.010 ***	0.010 ***
	No. Children		0.056 **	0.057 **	0.057 **
	Confirmed		0.190	0.209	0.194
	Deaths		-0.292	-0.300	-0.306

Each column (such as SC) represents the results of a fixed-effects regression model that includes the corresponding policy as a dummy variable. The p-value for the F-test across the three regression models is 0, and all the reported overall R-squared values are 0.05. The response to the life satisfaction question is the dependent variable (SWB-ladder). The data points were collected by a Gallup Panel COVID-19 survey from March 13, 2020 to Aug 30, 2020. During this period, some policies, such as SC, were directly adjusted from level 0 to level 2 or a higher level. Hence, we only include the SWB effects for specific stringency levels in the table. For dummy variables with several categories, such as the state, we only report the state with the most negative coefficient (NH for state) and the most positive effects (MI for state). The reference level for each categorical and dummy variable is also indicated (e.g., *Compared to female* for gender).

*, **, and *** indicate significance at the 90%, 95%, and 99% levels respectively. The P-values of the F-test for these models are indistinguishable from 0, making the overall regression highly significant.

**Table 3:** Fixed-effects regression results of the gathering restrictions (GR), transport closing (TC), and stay at home restrictions (SHR) policies.

			GR	TC	SHR
Dummy Variables	Policy (Compared to Level 0)	Level 1	1.300 *	-0.127	-
		Level 2	0.207	-0.178	-0.005
		Level 3	0.161	-	-
		Level 4	0.205	-	-
	Gender (Compared to Female)	Male	0.210 ***	0.210 ***	0.210 ***
	Employed Situation (Compared to Full-time)	Part-time	-0.083	-0.082	-0.083
	Job Area (Compared to Low-affected Jobs)	Middle-affected	0.157 **	0.156 **	0.156 **
		High-affected	0.094	0.094	0.094
	Has Young Children (Compared to No)	Yes	-0.171 **	-0.174 **	-0.173 **
	Has Older Children (Compared to No)	Yes	-0.186 **	-0.186 **	-0.186 **
	Income (Compared to Low-income)	Middle-income	0.560 ***	0.564 ***	0.560 ***
		High-income	0.884 ***	0.888 ***	0.884 ***
	Party (Compared to Democrat)	Republican	0.286 ***	0.286 ***	0.285 ***
	State (Compared to AK)	NH	-0.218	-0.127	-0.424
		MI	1.005	1.286	1.236
Numeric Variables	Age		0.010 ***	0.010 ***	0.010 ***
	No. Children		0.056 **	0.056 **	0.056 *
	Confirmed		0.211	0.198	0.192
	Deaths		-0.289	-0.305	-0.294

*, **, and *** indicate significance at the 90%, 95%, and 99% levels respectively. The P-values of the F-test for these models are indistinguishable from 0. All the reported overall R-squared values are 0.05. Other details are similar as noted earlier for Table 2.

Unlike the positive partial correlation between the policy and the measure of life satisfaction mentioned before, we find that when the socio-demographic variables are taken into consideration, the tightening of the SC policy caused a significant decrease in people’s life satisfaction. When the SC policy is adjusted from the lowest stringency measure (‘no measures’ or level 0) to level 3, the response to the *SWB-ladder* question shows an average decrease of 1.422, controlling for all socio-demographic variables. While the decrease in life satisfaction is considerable, it is not significant at the 90% confidence level. This decrease can only be compensated by the increase in the fixed effects of male respondents engaging in professional services with high annual household incomes.

The data also shows that the stringent WC, TC, and SHR policies have negative impacts on people’s SWB. The first three were also confirmed to be negatively associated with SWB in the correlation analysis. For the TC policy, we find that when the government adjusted it from level 0 to level 2, an average but insignificant decrease of 0.178 (*P* ≈ 0.5) is observed in people’s life satisfaction. The decrease is much smaller compared to the decrease caused by SC and gets reversed for male respondents.

Surprisingly, the stringent GR policy has a positive impact on the SWB of the participants, especially when the policy is just slightly tightened. When the policy is adjusted from level 0 to level 1, for instance, there is an average increase of 1.3 (*P* = 0.056) in people’s life satisfaction. However, a more stringent policy reduces this increase to 0.205 (*P* = 0.215) when the policy is adjusted from level 0 to level 4.

In considering the socio-demographic variables, we find that, although the estimated effect of the number of children in a household is positive (around 0.056; *P* = 0.014), having children in the home (especially with those under 12) has a negative effect on people’s life satisfaction (with fixed-effect coefficient of around -0.171; *P* = 0.037). In theory, a family needs at least 3 children to make up for the decline in parents’ life satisfaction caused by the fixed effects of having young children in the household. Families with older children (children aged 12–17) tend to report slightly lower levels of life satisfaction than those with younger children.

The fixed effects of states that respondents lived in varied across the regression model results. However, it was consistently found that the participants from Michigan, a Democratic state, tended to have the highest life satisfaction compared to participants in other states. Participants from New Hampshire, a state often recognized for its moderate politics, tended to provide the lowest SWB assessments.

The mean effect of the Republican dummy variable is around 0.286 (*P* < 0.01). Although in the previous correlation analysis, we did not observe that there is a significant difference in life satisfaction between Democrats and Republicans, the regression results show that the SWB assessment during the early period of the pandemic do strongly correlate with the political affiliation.

The job area and household income level of the respondents also play important roles in the assessment of their current life satisfaction. Respondents engaged in jobs that are moderately affected by the pandemic, such as government, public policy, and education tend to give the highest ratings of their SWB (a significant increase of 0.156; *P* < 0.01), but those employed in job areas highly impacted by COVID-19, such as hospitality and retail, generally rated their current living conditions similar to those engaging in low-affected jobs (with fixed effects around 0.1). Compared to the lowest income group, people at higher household income levels are more likely to be satisfied with their life and the strongest difference (with fixed effects around 1.0; *P* < 0.01) is observed in participants with over $180,000 household income per year, as compared to those making (at most) $48,000 annual household income.

Finally, because the conclusions remain robust when considering other related policies such as internal movement restrictions (IMR), testing policy (TP), and contact tracing (CT) policies, we reproduce them in S14 Table in S1 File.

### Objective 2 (predicting individual SWB using a conditional inference tree)

**Fig 2:**
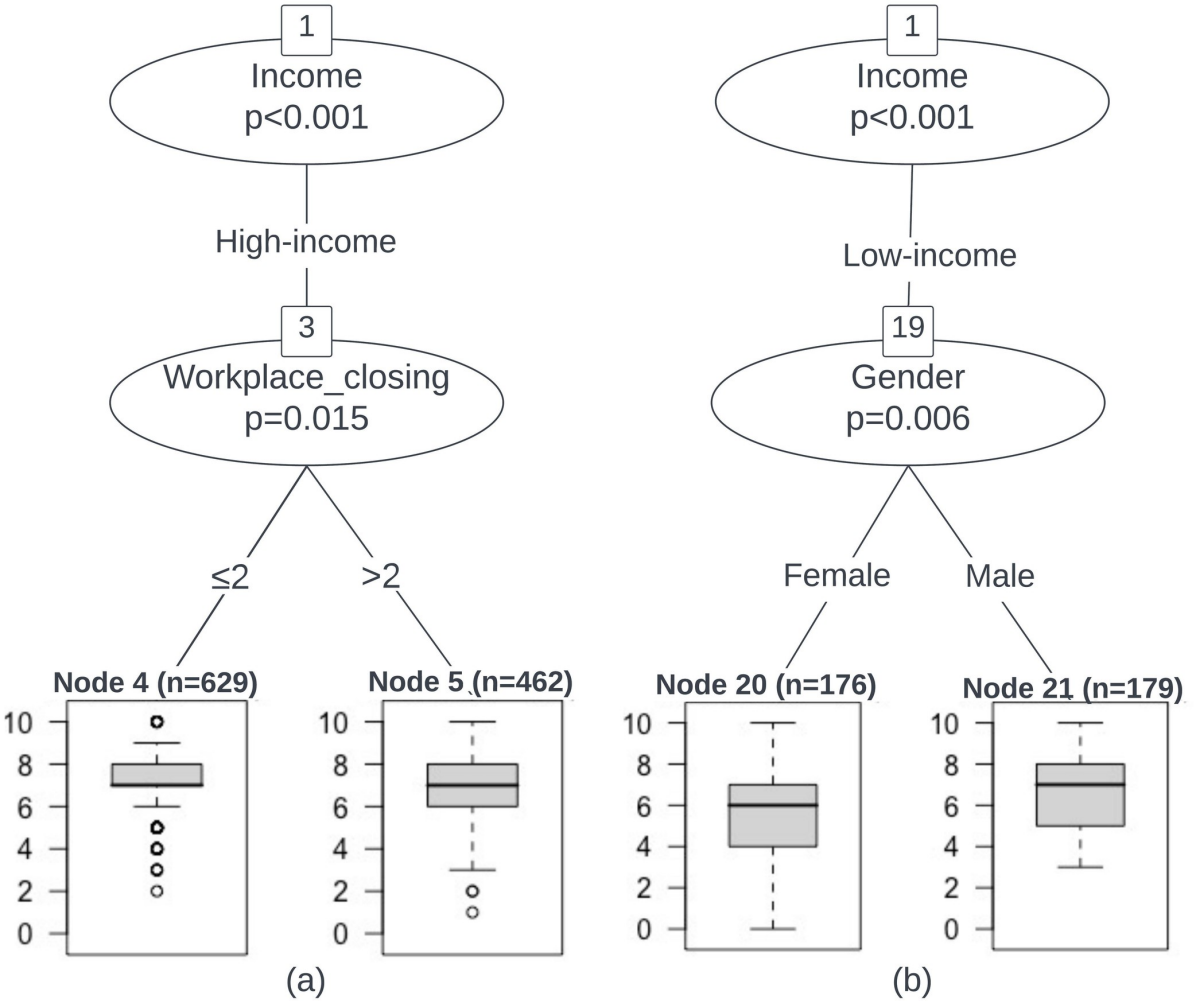
A visualization of the conditional inference tree (CIT) model for predicting life satisfaction (SWB-ladder) specifically for individuals in the high-income (a), and low-income groups (b). The tree shows only variables significant at the 90% level or higher. The total sample size (N) for the life satisfaction responses is 9,285, with half used for training, and half for testing / validation.

**Fig 3:**
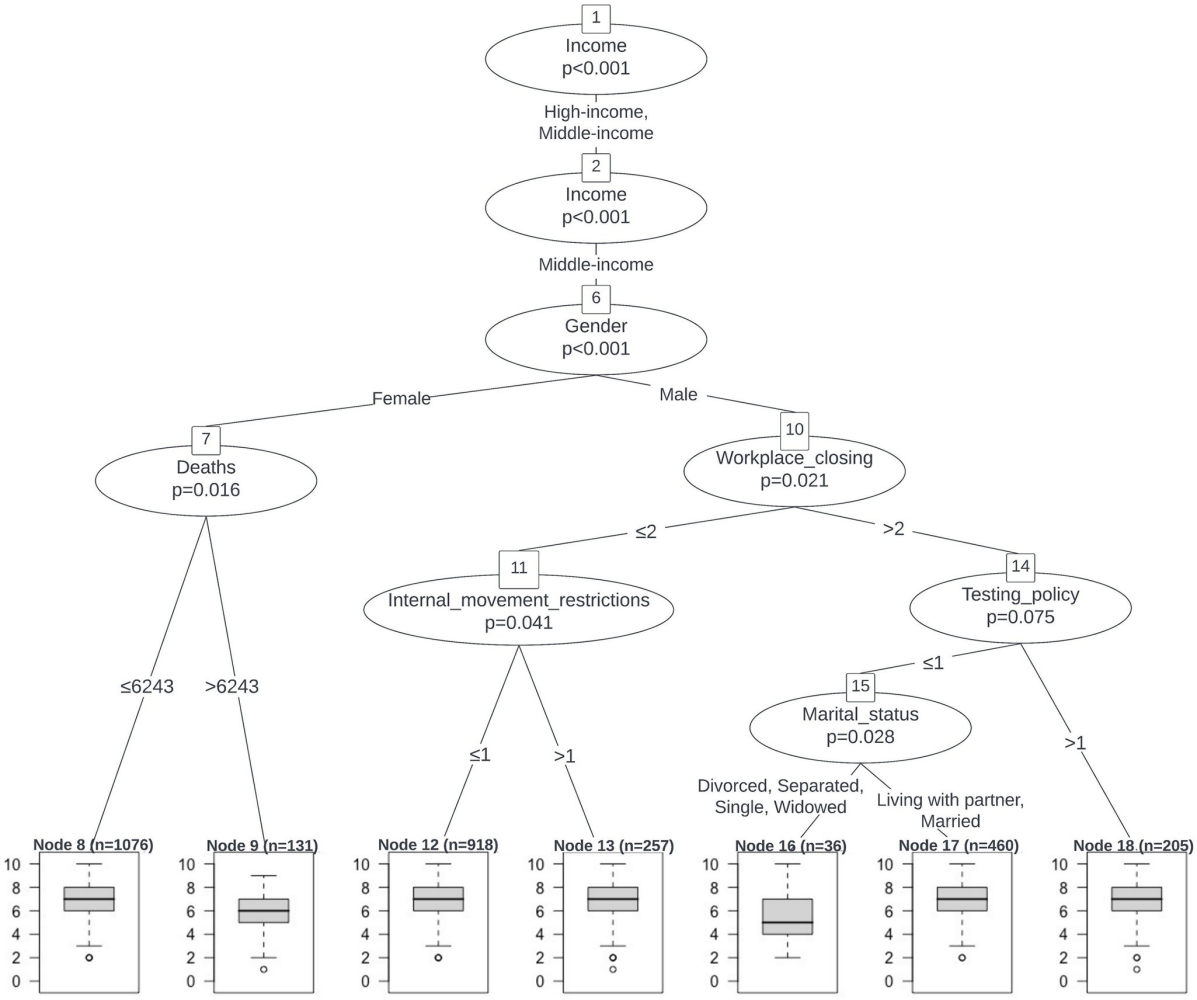
A visualization of the conditional inference tree (CIT) model for predicting life satisfaction (SWB-ladder) specifically for individuals in the middle-income group. The tree shows only variables significant at the 90% level or higher. The total sample size (N) for the life satisfaction responses is 9,285, with half used for training, and half for testing / validation.

The earlier results showed that the containment and closure policies such as WC and IMR have significant negative effects on individuals’ SWB as reported using the life satisfaction measure (SWB-ladder). In this section, we aim to determine the conditional and interactive effects between these policy variables and socio-demographic variables on SWB, by building a predictive model based on the conditional inference tree (CIT). The trained CIT model is both interpretable (and is visualized in Figs 2 and 3) and also robust, achieving a low mean squared error of 2.32 on the test set and 2.19 on the training set.

As shown in Figs 2 and 3, the annual household income level, gender, and the WC policy are the most important and significant predictors. Female individuals with low-income suffered significantly more than other groups, which has since been borne out with other supporting commentary from the media and related sources [55]. Their median life satisfaction was only around six, and is consistent with the regression results where we found a common increase in life satisfaction when comparing men to women, and high- and middle-income to low-income groups (with fixed values for the other variables). The effect of gender is strong enough that, even among middle-income women, when the daily death toll caused by COVID-19 exceeded 6,243, median life satisfaction decreased to six.

For participants with high incomes, varied responses to SWB are observed for the WC policy. As shown in Fig 2, the median life satisfaction is close to seven regardless of whether the stringency level of the policy is higher than two. However, when the stringency level is low, responses become more unevenly distributed.

The factors affecting the life satisfaction of the middle-income group are more complex. Except for variables such as gender and WC stringency, the TP and IMR policies also have significant effects on SWB. More ‘outliers’ (with very low values) are observed in the responses of the middle-income group (on life satisfaction) when the corresponding policies became more stringent. For middle-class men, when the WC policy is stringent but the TP policy is not, those who lived with a partner or were married, reported significantly higher SWB scores than those who were single. For instance, the median life satisfaction of middle-income single men is even lower than that of low-income women.

## Discussion

Before discussing our findings in a broader context, we highlight three key findings from the study:

1. Results from the fixed-effects regressions confirm that both the state and socio-demographic covariates significantly affected the association between policy impacts and SWB. This is most apparent when we consider the *School Closure (SC)* policy. In contrast with the positive partial correlation between SC and SWB, when the socio-demographic variables are taken into consideration, the tightening of the SC policy causes a significant decrease in people’s life satisfaction. Therefore, socio-demographic controls are important to include in any such model that aims to measure public sentiment toward policy impacts.
2. Compared to ‘low affected’ jobs, we found that a policy’s impacts on moderate or ‘middle affected’ jobs tended to be more significant (and usually positive) than the ‘highly affected’ jobs, which tended to be positive, but insignificant. We consistently observe this for the SC, WC, CE, GR, TC and SHR policies, among others. Thus, there are complex factors at play when it comes to quantifying a policy’s impacts on a person occupied in a given sector.
3. Similarly, the CIT model suggests that the factors affecting SWB for the *middle-income* group are also more complex. Considering middle-income men, for example, we found that when the WC policy is stringent but the TP policy is not, those who lived with a partner or were married, reported significantly higher SWB scores than those who were single. The median life satisfaction of middle-income single men was found to be even lower than that of low-income women.

Although these are not the only findings resulting from this study, we focus on them as they yield some key points of interest. The first finding has an important methodological implication. Partial correlations, while good for exploratory purposes or preliminary analysis, can result in misleading conclusions if only one socio-demographic variable (such as income or political affiliation) is controlled for. When more such variables are taken into account, a different relationship can emerge, or one that is not always statistically significant. For example, compared to the least stringent level (Level 0) of some policies like TC and SHR, there was no impact on SWB at higher levels of stringency (once socio-demographic controls and fixed effects are accounted for), insofar as we can observe in the recorded data. For policies like SC, we do observe a negative coefficient at a higher stringency level (Level 3) but it is not significant even at the 90% level.

Concerning the second finding, we hypothesize that the issuance of stimulus to US households (by the federal government) even in the early phase of the pandemic may explain why people employed in moderately affected sectors experienced a gain, rather than a loss, in well-being even though they likely suffered some loss of job-related income (especially in sectors like construction) when closures and containment policies were in place. At least one set of authors has recently found some evidence that this may indeed be the case [56]. However, it is difficult to isolate the impacts of stimulus directly in our study, as they were issued at the federal level (and even if there were heterogeneity, Gallup did not solicit this data on their survey). However, the hypothesis is, by no means, settled, and other datasets may be needed to understand this complex issue further.

Finally, considering the third finding, there has now been a consistent body of literature showing that men have been experiencing loss of SWB for many years now, well before the pandemic [57, 58]. Middle-income men are also likely to be employed in jobs that were moderately affected by the pandemic, which may be leading to effect modification that is not measured by the regression. However, the CIT model leads to a similar conclusion, making it unlikely that effect modification is the only, or even the dominant, explanation underlying this finding. Regardless of the actual cause (on which more research is clearly needed), we do note that the gender-based differences yielded by our models can serve as useful guides in future pandemic preparedness and policy design efforts. For example, in parts of the country that are predominantly middle-income and where men are employed in occupations moderately affected (by the pandemic), policy makers will want to question whether containment policies that they design have a higher likelihood of success or compliance or, if instead, they lead to resentment and populism. An alternative way to view the finding may be as a reflection of rising levels of income inequality in the country (only aggravated by COVID-19), by which the middle-class has been argued to have been particularly affected or ‘hollowed out’ [59, 60].

While some patterns are consistent, more broadly, the results in the previous section showed that the relationship between SWB, socio-demographics and policy stringency levels are complex. First, the importance of incorporating socio-demographic variables in research involving measurements of public SWB is emphasized in the finding (among others) that there may be class differences in the SWB responses to policies. Specifically, we found that low-income groups tend to have different SWB responses compared to high-income and middle-income groups. One policy where this difference was found to be most stark was workplace closure (WC). Since essential services were still open under this policy, low-income groups were not as worried as higher-income groups; however, this finding underscores the fact that essential services are performed by low-income groups. Over time, as the pandemic progressed, this may have been responsible for excessively burdening essential workers (especially among racial and ethnic minorities [61]), although it is not detectable in our data due to the time period in which we conducted our analyses.

### Comparing the findings to studies in other global and regional contexts

In comparing our findings with others that have since been published in the context of other regions, we find evidence in some other countries that policy implementation could have been more coordinated and effective. Knaul et al. present a new concept called *punt politics* and using a novel dataset, apply it to understand certain effects of COVID-19 non-pharmaceutical interventions in Mexico and Brazil [62]. They find that the “fragmentation of authority and overlapping functions in federal, decentralized political systems” can lead to coordination problems and reduced “health system functionality.” The policies that we study in this article may also be considered to be (largely) ‘non-pharmaceutical interventions’ and within the United States, were implemented at different levels of stringency in different states. Both our study, and the one in [62], show that such interventions are clearly important, and have implications beyond just health.

Another study [34], also conducted in Brazil, was structurally similar to ours in that the authors collected daily information on implementation of 10 non-pharmaceutical interventions across Brazil’s 27 states; however, their goal was to present an analysis of sub-national variation in implementation (which was found to be significant, and associated with the political affiliations of the states’ governors), rather than to measure public sentiment through SWB measures. However, their data could potentially be combined with proxy measures of SWB (if available) to replicate an analysis similar to our own, but specifically for Brazil. Methodologically, both their study and ours could be used retrospectively to understand variation in, and the SWB effects of, containment policies and other non-pharmaceutical interventions in a specific region or country. Other relevant studies that are methodologically similar, or have similar goals, include [63, 64]; outside of the Americas, studies on the impacts of COVID-19 containment policies include [65–68].

Castro et al. [69] analyzed the pattern of spread of COVID-19 (cases and deaths) in Brazil in the pre-vaccination phase, with the time period under study being very similar to our own. They found that disease spread was “accelerated by stark local inequalities and political upheaval, which compromised a prompt federal response.” Our results show that socio-demographics significantly affected policy impacts, and other studies suggest that they may have played a role in government distrust and vaccine hesitancy [27]. In particular, our results show that different job areas, genders and income groups were affected to varying extents (in terms of their SWB) by the patchwork of policies in place. In implementing such policies in the future, such factors should not be ignored in any assessment of expected compliance with the policies, especially among different socio-demographic segments.

We note that use of SWB in such studies is still relatively rare, perhaps because individual data on SWB is not made freely available by organizations like Gallup. Examples of such studies using SWB to better understand issues like COVID-19 vaccine hesitancy, conspiracy theories, and food insecurity, sometimes by leveraging external sources such as social media platforms and government datasets, include [70–73]. Promisingly, datasets (at least on the social media front) underlying some of these studies have been made available [74], which makes it possible, in principle, to build a joint model incorporating policy restrictions, SWB and vaccine hesitancy by drawing on diverse datasets.

### How do the findings inform future pandemic preparedness?

An important motivation underlying the study of COVID-19 policy impacts on SWB, even after many of the restrictions have been listed, is that it could help inform more effective pandemic preparedness efforts in the future [75, 76]. Even before COVID-19, a robust body of literature had been generated both on pandemic preparedness, and more broadly, crisis management; see e.g., [77–80], although COVID-19 has led to significant additional work in this area, given the global scope of the pandemic [81]. Researchers have been especially interested in vaccination, and policies to increase the rapid development and subsequent uptake of vaccines when a new pandemic breaks out [82, 83]. However, it is also evident that vaccination alone is not sufficient to contain a pandemic [84, 85]. Because vaccination became available relatively quickly (at least, by historical standards) after the pandemic spread, it is difficult to control for its effects when measuring the impacts of containment policies on SWB. Within the United States, due to intense political partisanship during the November 2020 presidential election (as evidenced through incidents such as the January 6 Capitol riots), it became increasingly difficult to tease apart policy impacts on SWB after controlling for political affiliation and vaccine hesitancy. We noted earlier that this was one of the reasons we chose to focus on the pre-vaccination phase of the pandemic (which in the US, also happened to be the pre-election period).

Our results show that public policy around pandemic containment, even without such confounding factors, need to minimally take into account the socio-demographic characteristics of the populace that is subject to such policies. Furthermore, not all policies are equally popular (or unpopular), and a priori, it may be difficult to predict the differential impacts on SWB. This suggests that the administrative arms of the government, including agencies like the Centers for Disease Control and Prevention (CDC) in the United States, need to take a more nimble and adaptive approach in the future rather than only rely on epidemiological models that predict health outcomes, but without consideration for deeper social implications. Recent technological advancements, including rapid electronic-based surveying, and availability of open-source artificial intelligence and computational social science tools, may serve as promising mechanisms to achieve such adaptiveness and flexibility at low cost [86–88].

One of the key findings in investigating the first objective was that significant differences were observed in correlations when controlling for the job area variable. More stringent policies were found to be positively associated with positive affect measures for people working in professional services. Retail, in contrast, was one of the most vulnerable industries affected by the pandemic. Workplace closure and internal movement restriction policies significantly reduced the SWB of retail workers, with economic effects likely playing a mediating effect. In other results of interest, the stringency level of the contact tracing policy had a significant and positive association with the worry measure for social workers. The reverse was observed for technology workers.

These differences, now more apparent, could have important implications for future pandemic preparedness. Some policies, when implemented stringently, can cause significant loss in SWB. While difficult to make a precise causal connection, such loss may contribute to subsequent loss of trust in public institutions. This loss of trust has been well documented now in the United States, although a number of causes for it have been postulated by social scientists [89–91]. The reason that this is important is that, once public trust is lost, obtaining compliance in the event of a future pandemic will be more difficult. Therefore, pandemic preparedness requires a more nuanced cost-benefit analysis that goes beyond (reduction in) the death and infection counts as the only objectives of a stringent containment policy. In his encyclopedic article, Coccia explicitly mentions that pandemic preparedness should not just be focused on the health outcomes of policies, but also consider “negative impact in society” and include the design of strategies to support different, and potentially more effective, policy responses [81].

Gender-based differences were also found to be important, and are congruent with other findings in the literature [92]. For instance, in investigating the second objective, we found that, when the daily death toll caused by COVID-19 exceeded 6,243, median life satisfaction among middle-income women declined to six. The CIT also showed that the effect of gender was mediated (at least in part) by the COVID-19 impact variables. Marital status was also important: for middle-income men, when the WC policy is stringent but testing policies are not, those married or living with a partner generally reported significantly higher life satisfaction than those who were single. One way to interpret this result (albeit not the only one) is that marriage may have helped alleviate the loneliness that was caused due to the social distancing protocols, and closures of schools and workplaces, implemented during the pandemic. Other results, including a meta-analysis, have since confirmed that loneliness was a significant problem during the pandemic [93–95].

Regression analyses were largely confirmatory in nature, but more nuances were observed across the policy measures. For example, while the restrictions on gatherings (GR) policy had a positive impact on SWB when it is just slightly tightened (from level 0 to level 1), its effect is non-monotonic. Compared to an average increase of 0.71 (*P* = 0.036) when going from level 0 to 1, the increase is only 0.044 (and is not distinguishable from 0 at a confidence level of 90 percent or above) when going from level 0 to level 4. This result further demonstrates that our decision not to treat a policy’s stringency level as an ordinal real-valued variable was appropriate. Instead, these levels should continue to be treated as categorical to detect such non-monotonic impacts.

The conditional inference tree (CIT) model achieved a low mean squared error of 2.32 on the test set and 2.19 on the training set. The income status, gender and the workplace closure (WC) policy were found to be the most important and significant predictors of life satisfaction. We noted some earlier effects of gender and marital status earlier. More broadly, our methodology shows that the CIT model offers a feasible way to model complex dependencies in a statistically rigorous manner. Recently, it was also applied to quantify the influence of socio-demographic covariates on vaccine hesitancy [27]. In future work on pandemic preparedness, it could be used to rapidly analyze survey data to build predictive models that can be used to implement more targeted (and effective) policies.

## Limitations

Certain limitations characterize the study. First, we note that the use of SWB is itself subject to some limitations and caveats. Diener states that while (as a general rule) SWB measures tend to “correlate moderately with each other and have adequate temporal reliability and internal consistency” [8], multi-item scales have still not been tested as rigorously and that a greater connection is needed between theory and practice in SWB research.

Furthermore, because the pandemic was a unique episode of our time, it is also likely that there are other factors that contributed to the results but that were not included in either the Gallup or the OxCGRT data. One important set of factors that bears noting here is the ‘misery’ factors such as food insecurity, unemployment, and not being able to cover basic needs, which are all known to cause significant declines in SWB [96, 97]. Some of these factors, especially unemployment, are known to widely affect the US population, even pre-COVID [98, 99]. Based on a report from the United States Department of Agriculture, food insecurity in America affected as many as one in nine Americans (an estimated total of over 37 million Americans) even in pre-COVID days [100]. Post-COVID, the situation took a turn for the worse, with long lines at food banks and pantries considered one of several defining images capturing the toll the crisis has taken on American families [101]. In an auxiliary study that we conducted using another data source [71], we found that misery variables indeed show negative correlation with the positive affective SWB measures. Although such variables are generally associated with income, which was one of our variables in the main results, the correlation is not perfect. Hence, there is always the possibility that, in a more complete study, some of the weaker or less significant associations reported in this study could be reversed or become insignificant. Conversely, with better controls, it is equally possible that some of our less significant findings could be strengthened. In either case, the conclusions could change, but this is a general limitation of such observational studies.

Two other factors that might limit the generalizability of this study for future pandemics or health emergencies are political polarization, which has arguably intensified in the period following our analysis owing to events such as the Jan. 6 Capitol riots, and increasing distrust from some segments of the population concerning both public health measures and vaccines. In at least one study, vaccine hesitancy has been found to have a complex association with SWB [72]. Vaccine hesitancy has proven to be a difficult challenge to resolve, and not just for COVID-19 [102, 103]. Yet another confounder in later periods is varied adherence to ‘official’ policies in different communities [104]. All of these factors have to be taken into account when attempting to replicate such a study in other contexts (including other nations and emergent public health situations).

One other limitation that we had briefly alluded to in the introduction, but that is worth emphasizing again, is that causality cannot be inferred from a study of this nature, as the models used are necessarily associative by design. Although SWB was modeled as a dependent variable in investigating the first objective, as well as the predictive goal of the second objective, changes to it cannot definitively be said to have been ‘caused’ by the policies. Similarly, we cannot interpret the CIT as a causal tree where a parent node in the tree is a ‘cause’ of the child. That being said, our findings do show that a quantifiable and significant association can be detected between some policy variables and SWB measures, even after controlling for established socio-demographic drivers of SWB. Furthermore, ‘reverse’ causality can also be reasonably discarded as a possibility: changes in SWB do not cause policy stringency levels to change, as these are not variables that the general public is free to manipulate over a short period of time (even if it were, we note that no elections took place in the 6-month period under study). Hence, studies of this kind can serve as important public health tools for conducting a cost-benefit analysis of pandemic preparedness and prevention policies that takes a more nuanced and comprehensive view of policy effectiveness e.g., their political and social implications, rather than just the health implications.

## Supporting information

S1 File (PDF)
