# Supplementary material for: Quantifying COVID-19 policy impacts on subjective well-being during the early phase of the pandemic: A cross-sectional analysis of United States survey data from March to August 2020
Source: PLoS One. 2023 Sep 21;18(9):e0291494. doi: 10.1371/journal.pone.0291494 (PMC10513291; doi:10.1371/journal.pone.0291494)
Supplement: S1 File — (PDF) [file pone.0291494.s001.pdf]

S1 Table. Checklisted STROBE Statement.

	Item No	Recommendation
**Title and abstract**	1	(*a*) ~~Indicate the study's design with a commonly used term in the title or the abstract~~
		(*b*) ~~Provide in the abstract an informative and balanced summary of what was done and what was found~~
**Introduction**		
Background/rationale	2	~~Explain the scientific background and rationale for the investigation being reported~~
Objectives	3	~~State specific objectives, including any prespecified hypotheses~~
**Methods**		
Study design	4	~~Present key elements of study design early in the paper~~
Setting	5	~~Describe the setting, locations, and relevant dates, including periods of recruitment, exposure, follow-up, and data collection~~
Participants	6	~~*Cross-sectional study*—Give the eligibility criteria, and the sources and methods of selection of participants~~
Variables	7	~~Clearly define all outcomes, exposures, predictors, potential confounders, and effect modifiers. Give diagnostic criteria, if applicable~~
Data sources/ measurement	8*	~~For each variable of interest, give sources of data and details of methods of assessment (measurement). Describe comparability of assessment methods if there is more than one group~~
Bias	9	~~Describe any efforts to address potential sources of bias~~
Study size	10	~~Explain how the study size was arrived at~~
Quantitative variables	11	~~Explain how quantitative variables were handled in the analyses. If applicable, describe which groupings were chosen and why~~
Statistical methods	12	~~(*a*) Describe all statistical methods, including those used to control for confounding~~
		~~(*b*) Describe any methods used to examine subgroups and interactions~~
		~~(*c*) Explain how missing data were addressed~~

		~~(*d*)*Cross-sectional study*—If applicable, describe analytical methods taking account of sampling strategy~~
		(*e*) Describe any sensitivity analyses
**Results**		
Participants	13*	(a) ~~Report numbers of individuals at each stage of study—eg numbers potentially eligible, examined for eligibility, confirmed eligible, included in the study, completing follow-up, and analysed~~
		(b) Give reasons for non-participation at each stage
		(c) Consider use of a flow diagram
Descriptive data	14*	~~(a) Give characteristics of study participants (eg demographic, clinical, social) and information on exposures and potential confounders~~
		(b) Indicate number of participants with missing data for each variable of interest
Outcome data	15*	~~*Cross-sectional study*—Report numbers of outcome events or summary measures~~
Main results	16	~~(*a*) Give unadjusted estimates and, if applicable, confounder-adjusted estimates and their precision (eg, 95% confidence interval). Make clear which confounders were adjusted for and why they were included~~
		~~(*b*) Report category boundaries when continuous variables were categorized~~
		(*c*) If relevant, consider translating estimates of relative risk into absolute risk for a meaningful time period
Other analyses	17	~~Report other analyses done—eg analyses of subgroups and interactions, and sensitivity analyses~~
**Discussion**		
Key results	18	~~Summarise key results with reference to study objectives~~
Limitations	19	~~Discuss limitations of the study, taking into account sources of potential bias or imprecision. Discuss both direction and magnitude of any potential bias~~
Interpretation	20	~~Give a cautious overall interpretation of results considering objectives, limitations, multiplicity of analyses, results from similar studies, and other relevant evidence~~

Generalisability	21	Discuss the generalisability (external validity) of the study results
**Other information**		
Funding	22	Give the source of funding and the role of the funders for the present study and, if applicable, for the original study on which the present article is based

S2 Table. The stringency level of COVID-19 policy measures. Descriptions quoted from OxCGRT codebook accessed at https://github.com/OxCGRT/covid-policy-tracker/blob/master/documentation/codebook.md

Policy	Description of stringency level
School Closure	0 - No measures taken
	1 - Recommend all schools open with adjustments that are significantly different from pre-COVID-19 (e.g., switch to a Hybrid Classroom)
	2 - Require the closure of certain levels or categories of schools (e.g., high school, or public schools)
	3 - Require closing schools at all levels
Workplace closing	0 - no measures
	1 - recommend closing (or recommend work from home) or all businesses open with alterations resulting in significant differences compared to non-Covid-19 operation
	2 - require closing (or work from home) for some sectors or categories of workers
	3 - require closing (or work from home) for all-but-essential workplaces (eg grocery stores, doctors)
Cancel events	0 - no measures
	1 - recommend cancelling public events
	2 - require cancelling public events
gatherings restrictions	0 - no restrictions
	1 - restrictions on very large gatherings (the limit is above 1000 people)
	2 - restrictions on gatherings between 101-1000 people
	3 - restrictions on gatherings between 11-100 people
	4 - restrictions on gatherings of 10 people or less
transport closing	0 - no measures
	1 - recommend closing (or significantly reduce volume/route/means of transport available)
	2 - require closing (or prohibit most citizens from using it)
stay home restrictions	0 - no measures
	1 - recommend not leaving house
	2 - require not leaving house with exceptions for daily exercise, grocery shopping, and 'essential' trips
	3 - require not leaving house with minimal exceptions (eg allowed to leave once a week, or only one person can leave at a time, etc)
internal movement restrictions	0 - no measures
	1 - recommend not to travel between regions/cities
	2 - internal movement restrictions in place

testing policy	0 - no testing policy
	1 - only those who both (a) have symptoms AND (b) meet specific criteria (eg key workers, admitted to hospital, came into contact with a known case, returned from overseas)
	2 - testing of anyone showing Covid-19 symptoms
	3 - open public testing (eg "drive through" testing available to asymptomatic people)
contact tracing	0 - no contact tracing
	1 - limited contact tracing; not done for all cases
	2 - comprehensive contact tracing; done for all identified cases

S3 Table. Summary statistics of the SWB-ladder variable, and each of the ten affective well-being variables.

Variable	Mean	Min	Max	Std Dev	Skewness	Kurtosis coefficient
SWB-ladder	6.89	0.00	10.00	1.57	-0.75	0.69
Enjoyment	0.69	0.00	1.00	0.46	-0.81	-1.35
Worry	0.45	0.00	1.00	0.50	0.19	-1.97
Sadness	0.26	0.00	1.00	0.44	1.12	-0.75
Stress	0.54	0.00	1.00	0.50	-0.16	-1.98
Anger	0.24	0.00	1.00	0.43	1.24	-0.47
Happiness	0.73	0.00	1.00	0.44	-1.06	-0.87
Boredom	0.28	0.00	1.00	0.45	0.96	-1.08
Loneliness	0.18	0.00	1.00	0.39	1.65	0.71
Depression	0.15	0.00	1.00	0.36	1.96	1.83
Anxiety	0.36	0.00	1.00	0.48	0.56	-1.69

S4 Table. Summary statistics of socio-demographic and COVID-based variables and their levels in the pre-processed dataset.

	Variable	Level	Mean	Min	Max	Std Dev	skewness	kurtosis coefficient
Numeric Variables	Age	-	51.65	20.00	105.00	12.08	-0.18	-0.55
	Number of children in household	-	0.68	0.00	10.00	1.08	1.89	4.72
	Confirmed COVID-19 cases per day	-	73460	915	392930	85945	2.37	5.61
	COVID-19 deaths per day	-	3880	7	24842	5596	2.67	0.69
Categorical Variables	Gender	Female	0.42	0.00	1.00	0.49	0.33	-1.89
		Male	0.58	0.00	1.00	0.49	-0.33	-1.89
	Employed Situation	Full-time paid worker	0.82	0.00	1.00	0.38	-1.67	0.78
		Part-time paid worker	0.18	0.00	1.00	0.38	1.67	0.78
	Has young children in household	No	0.03	0.00	1.00	0.17	5.45	27.74
		Yes	0.27	0.00	1.00	0.44	1.06	-0.88
	Has older children in household	No	0.04	0.00	1.00	0.20	4.64	19.49
		Yes	0.25	0.00	1.00	0.44	1.13	-0.73
	Job	Low-affected	0.18	0.00	1.00	0.38	1.66	0.75
		Middle-affected	0.44	0.00	1.00	0.50	0.23	-1.95
		High-affected	0.38	0.00	1.00	0.48	0.52	-1.73
	Income	Low-income	0.11	0.00	1.00	0.31	2.50	4.24
		Middle-income	0.68	0.00	1.00	0.46	-0.79	-1.37
		High-income	0.21	0.00	1.00	0.40	1.45	0.12

		Democrat	0.56	0.00	1.00	0.50	-0.24	-1.94
	Party	Republican	0.44	0.00	1.00	0.50	0.25	-1.94

S5 Table. Distribution of respondents' job area

Collapsed Level	Job area	Percentage
	Health Care	10.96
	Hospitality	1.36
	Retail	3.70
	Manufacturing	6.10
	Professional Services	4.12
	Construction	3.75
High-affected	Transportation	3.46
	Finance	3.92
	Government or Public Policy	8.22
	Technological/Information Systems/Computer/Software/Mathematical	6.69
	K-12 Education	9.93
	College or University	6.03
Middle-affected	Community/Social Services	1.92
	Arts/Design/Entertainment/Sports/Media	2.69
	Real Estate	1.98
	Utilities	1.88
	Mining, Quarrying, Oil, and Gas Extraction	0.79
	Agriculture, Forestry, Fishing, and Hunting	1.77
	Warehousing/Logistics	1.10
	Training or Library	0.66
	Insurance	2.24
Low-affected	Law	3.30

S6 Table: The partial correlations between SWB variables and COVID-19 policy measures for Democrats and Republicans

**Democrats**											
	Ladder	Enjoyment	Worry	Sadness	Stress	Anger	Happiness	Boredom	Loneliness	Depression	Anxiety
school_closing	0.015	0.025 *	-0.011	-0.004	-0.022	-0.036 **	0.030 **	-0.011	-0.025 *	-0.059	-0.067 ***
workplace_closing	-0.082 ***	-0.051 ***	0.103 ***	0.068 ***	0.075 ***	0.027 *	-0.042	0.095 ***	0.017	0.014	0.065
cancel_events	-0.055 ***	-0.033 **	0.045	0.050 ***	0.042	0.023	-0.041	0.073 ***	0.014	0.045 **	0.047 **
gatherings_restrictions	-0.050 ***	-0.049 ***	0.071 ***	0.041	0.037	0.014	-0.029 **	0.023	0.039	0.050 **	0.044 **
transport_closing	0.001	-0.002	-0.034 **	-0.016	-0.006	0.003	-0.011	-0.011	0.005	-0.025	-0.025
stay_home_restrictions	-0.062 ***	-0.038	0.067 ***	0.046	0.062 ***	0.002	-0.033 **	0.096 ***	-0.001	-0.024	0.034 *
internal_movement_restrictions	-0.017	-0.013	0.029 **	0.014	0.014	-0.002	-0.006	0.036 **	-0.016	0.005	-0.007
testing_policy	0.047 ***	0.048 ***	-0.075 ***	-0.051 ***	-0.050 ***	0.006	0.035 **	-0.084 ***	-0.013	-0.001	-0.024
contact_tracing	0.02	0.008	0	-0.012	-0.007	0.031 **	0.01	-0.056 ***	-0.005	0.035 *	0.027

**Republicans**											
	Ladder	Enjoyment	Worry	Sadness	Stress	Anger	Happiness	Boredom	Loneliness	Depression	Anxiety
school_closing	0.012	-0.029 *	0.062 ***	0.025	0.053	0.015	-0.021	0	0.024	0.034	0.049 **
workplace_closing	-0.064 ***	-0.053	0.067 ***	0.047	0.077 ***	0.012	-0.05	0.087 ***	0.003	0.024	0.005
cancel_events	-0.034 **	-0.022	0.006	0.022	0.026	0.01	-0.021	0.083 ***	-0.026	-0.031	-0.006
gatherings_restrictions	-0.021	-0.019	0.024	0.025	0.045	-0.013	-0.007	0.060 ***	-0.038 **	-0.017	-0.003
transport_closing	0.004	0.001	-0.008	0.035 **	0.011	0.030 *	0.004	-0.004	0.02	-0.026	-0.017
stay_home_restrictions	-0.045	-0.036 **	0.016	0.029 *	0.032 *	0.01	-0.022	0.080 ***	0.011	-0.038	-0.012
internal_mo	-0.048	-0.044	0.030 *	0.034	0.058	0.018	-0.028	0.041	0.012	-0.036	-0.004

vement_restrictions				**	***		*	**			
testing_policy	0.080 ***	0.045	-0.066 ***	-0.028 *	-0.070 ***	-0.001	0.025	-0.080 ***	-0.009	0.027	0.014
contact_tracing	0.039 **	0.038 **	-0.007	-0.028 *	-0.026	0.002	0.024	-0.058 ***	0.005	0.027	0.017

S7 Table: The partial correlations between SWB variables and COVID-19 policy measures for low-income, middle-income, and high-income groups

Low-income											
	Ladder	Enjoyment	Worry	Sadness	Stress	Anger	Happiness	Boredom	Loneliness	Depression	Anxiety
school_closing	0.022	0.051	0.067	0.044	0.06	-0.015	0.029	0.04	0.012	0.071	0.091
workplace_closing	0.005	0.031	-0.029	-0.001	-0.031	0.042	-0.006	0.062	-0.146	0.053	-0.06
cancel_events	0.006	0.019	-0.065	0.035	-0.021	0.009	-0.077 *	0.111 **	-0.098 **	-0.013	0.037
gatherings_restrictions	0.034	0.004	-0.035	0.059	-0.025	-0.015	-0.008	0.051	-0.032	0.005	-0.002
transport_closing	-0.018	0.003	0.001	0.044	-0.045	0.049	-0.041	-0.082 *	-0.021	0.01	-0.136 **
stay_home_restrictions	-0.015	0.022	-0.018	0.006	-0.007	0.029	-0.027	0.053	-0.136	-0.133 **	-0.144 **
internal_movement_restrictions	-0.093 **	-0.01	0.002	0.028	0.007	0.016	-0.049	0.029	-0.014	0.109 *	0.031
testing_policy	0.043	-0.027	-0.015	-0.027	0.012	0.026	0	-0.05	0.069	0.03	-0.048
contact_tracing	0.102 **	-0.04	0.068	-0.021	0.066	0.051	-0.022	-0.117	0.038	0.044	0.105

Middle-income											
	Ladder	Enjoyment	Worry	Sadness	Stress	Anger	Happiness	Boredom	Loneliness	Depression	Anxiety
school_closing	0.038 *	0.004	0.001	-0.045 **	0.011	-0.044 **	0.02	0.001	-0.034 *	-0.04	-0.016
workplace_closing	-0.067 ***	-0.062	0.071 ***	0.078 ***	0.049 **	-0.009	-0.058	0.100 ***	0.018	0.014	0.056 *
cancel_events	-0.050 **	-0.045 **	0.061	0.051 **	0.032	0.02	-0.053 **	0.079 ***	0.017	0.041	0.045
gatherings_restrictions	-0.024	-0.047 **	0.036 *	0.023	0.045 **	-0.013	-0.032	0.050 **	0	0.023	0.051 *
transport_closing	0.012	0.012	-0.033	0.003	-0.005	0.008	0.01	0.024	-0.012	-0.046	0.006
stay_home_restrictions	-0.083 ***	-0.061	0.03	0.071 ***	0.035 *	0.005	-0.038 *	0.113 ***	0.015	0	0.023
internal_mo	-0.042	-0.058	0.014	0.038 *	0.018	0.017	-0.048	0.004	-0.003	-0.050	-0.037

vement_restrictions	**						**			*	
testing_policy	0.061	0.053 **	-0.062	-0.057	-0.053 **	0.017	0.03	-0.069 ***	-0.007	0.007	-0.047
contact_tracing	0.031	0.041 **	-0.011	-0.050 **	-0.028	0.025	0.046 **	-0.036 *	0.02	0.024	-0.029

High-income											
	Ladder	Enjoyment	Worry	Sadness	Stress	Anger	Happiness	Boredom	Loneliness	Depression	Anxiety
school_closing	0	-0.011	0.035	0.031 **	0.018	0.005	-0.006	-0.011	0.015	0.002	0
workplace_closing	-0.115 ***	-0.064 ***	0.112 ***	0.075 ***	0.101 ***	0.045 ***	-0.058 ***	0.091 ***	0.035	0.029	0.054
cancel_events	-0.061 ***	-0.027 **	0.023 *	0.036	0.041	0.019	-0.022 *	0.076 ***	0.002	0.009	0.012
gatherings_restrictions	-0.070 ***	-0.036	0.063 ***	0.046 ***	0.051 ***	0.019	-0.023 *	0.04	0.006	0.029	0.028
transport_closing	0.001	-0.008	-0.017	0.005	0.01	0.017	-0.01	-0.016	0.023 *	-0.02	-0.014
stay_home_restrictions	-0.063 ***	-0.038	0.062 ***	0.039	0.063 ***	0.011	-0.032 **	0.083 ***	0.021	-0.028	0.026
internal_movement_restrictions	-0.034	-0.019	0.044 ***	0.026 *	0.043	0.01	-0.007	0.052 ***	0	-0.011	0.006
testing_policy	0.076 ***	0.055 ***	-0.085 ***	-0.044 ***	-0.070 ***	-0.011	0.04	-0.093 ***	-0.027 **	0.001	0.003
contact_tracing	0.026 **	0.022 *	-0.011	-0.014	-0.02	0.007	0.012	-0.060 ***	-0.016	0.026	0.027

S8 Table: The partial correlations between SWB variables and COVID-19 policy measures for low-affected, middle-affected, and high-affected job groups

Low-affected jobs											
	Ladder	Enjoyment	Worry	Sadness	Stress	Anger	Happiness	Boredom	Loneliness	Depression	Anxiety
school_closing	-0.023	0.023	-0.059	0.03	-0.046	0.032	0.036	0.04	0.023	nan	nan
workplace_closing	-0.029	-0.03	0.103 **	0.095 **	0.107	0.082 **	-0.101 **	0.082 **	0.016	0.03	0.211 ***
cancel_events	0.005	-0.041	0.018	0.05	0.011	0.027	-0.021	0.072 *	0.041	-0.012	0.105 *
gatherings_restrictions	0.009	-0.022	-0.013	0.026	0.028	-0.007	-0.021	0.090 **	0.01	0.014	0.008
transport_closing	0	-0.046	-0.04	-0.045	-0.014	0.003	-0.043	0.105	0.056	-0.016	0.092
stay_home_restrictions	-0.059	-0.04	0.031	0.049	0.062	0.011	-0.012	0.121	-0.025	-0.001	0.129 **
internal_movement_restrictions	-0.018	0.057	0.062	0.055	0.041	0.051	0.012	-0.046	-0.034	-0.071	-0.013
testing_policy	0	0.049	0.005	-0.077 *	-0.024	-0.01	-0.01	-0.147 ***	0.033	0.007	-0.054
contact_tracing	0.031	0	-0.039	-0.082 **	-0.022	-0.023	-0.037	-0.072 *	0.012	0.058	0.024

Middle-affected jobs											
	Ladder	Enjoyment	Worry	Sadness	Stress	Anger	Happiness	Boredom	Loneliness	Depression	Anxiety
school_closing	-0.005	-0.026	0.001	-0.02	-0.029	-0.022	-0.021	0.026	0.015	0.015	-0.047
workplace_closing	-0.076	-0.053 **	0.074	0.088 ***	0.072	0.054 **	-0.015	0.063 **	0.052 **	0.082 **	0.091 **
cancel_events	-0.031	-0.044 *	0.006	0.041	0.038	0.026	-0.035	0.062 **	0.003	0.066 *	0.075 **
gatherings_restrictions	-0.060 **	-0.054 **	0.055 **	0.059 **	0.064 **	0.019	-0.021	-0.023	0.001	0.084 **	0.072 *
transport_closing	-0.08	-0.048 *	-0.003	0.052 **	0.025	0.036	-0.007	-0.018	0.019	-0.011	-0.017
stay_home_r	-0.067	-0.054	0.035	0.067	0.081	0.056	-0.028	0.086	0.021	0.008	0.06

estrictions		**				**		***			
internal_movement_restrictions	-0.012	-0.017	0.022	-0.016	0.01	-0.009	0.014	0.065 **	0.005	-0.035	-0.059
testing_policy	0.047 *	0.055 **	-0.007	-0.021	-0.034	-0.012	0.03	-0.043 *	-0.014	0.016	-0.01
contact_tracing	0.013	0.01	0.013	0.02	-0.008	0.029	0.005	-0.055 **	0.012	0.036	-0.014

High-affected jobs											
	Ladder	Enjoyment	Worry	Sadness	Stress	Anger	Happiness	Boredom	Loneliness	Depression	Anxiety
school_closing	-0.086	-0.093 ***	0.058 **	-0.026	0.042	-0.017	-0.077	0.073	0.02	0.002	0.027
workplace_closing	-0.037	-0.043	0.032	0.024	0.064 **	-0.043	-0.03	0.039	0.029	0.051	0.021
cancel_events	-0.065 **	-0.050 *	0.044	0.026	0.034	-0.016	-0.038	0.013	-0.013	0.054	0.048
gatherings_restrictions	-0.001	0.026	-0.033	0.001	0.003	0.035	0.004	-0.037	-0.003	-0.106	-0.033
transport_closing	-0.068 **	-0.079	0.036	-0.033	0.036	-0.013	-0.078	0.060 **	0.023	0.03	0.076 *
stay_home_restrictions	-0.069 **	-0.027	0.035	-0.03	0.061 **	0	-0.048 *	0.065 **	-0.011	0.032	0.063
internal_movement_restrictions	-0.034	-0.019	0.044 ***	0.026 *	0.043	0.01	-0.007	0.052 ***	0	-0.011	0.006
testing_policy	0.018	0.059 **	-0.054 *	0.050 *	-0.051 *	0.056 **	0.038	-0.041	0.006	-0.014	-0.018
contact_tracing	0.023	0.017	0.014	0.046	-0.036	0.012	0.021	-0.044	0.01	0.003	-0.051

S9 Figure: The partial correlation (y-axis) between Enjoyment variable and each COVID-19 policy measure (x-axis) after controlling for respondent's income status (a), party affiliation (b), and job areas (c). No correlation was observed between the school closing policy and the life satisfaction of respondents with job areas highly impacted by COVID-19 as the policy remained constant at level 3 across all corresponding data points. Error bars represent the standard errors of the correlation coefficients.

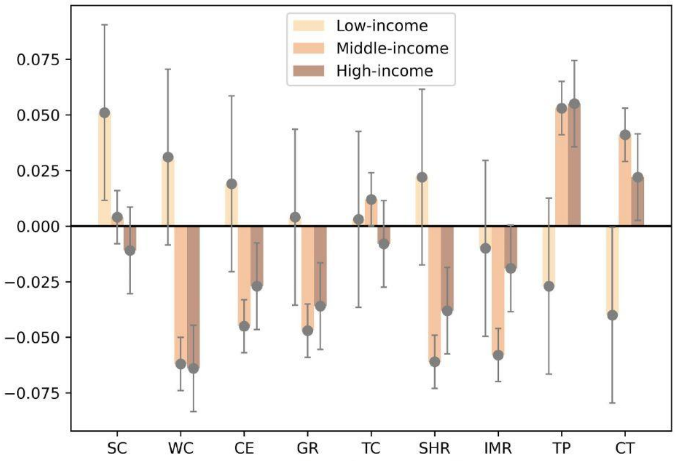

(a) Controling for income status

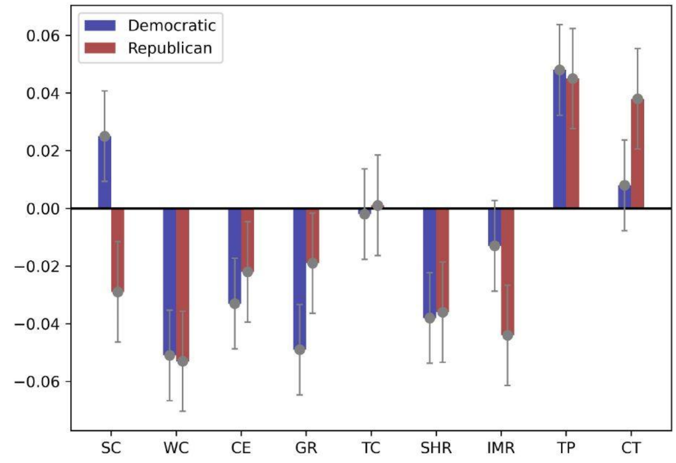

(b) Controling for party affiliation

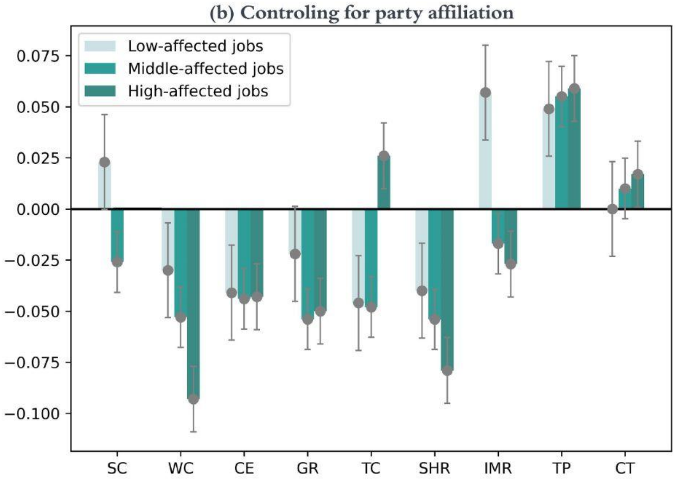

(c) Controling for job area

S10 Figure: The partial correlation (y-axis) between Worry variable and each COVID-19 policy measure (x-axis) after controlling for respondent's income status (a), party affiliation (b), and job areas (c). No correlation was observed between the school closing policy and the life satisfaction of respondents with job areas highly impacted by COVID-19 as the policy remained constant at level 3 across all corresponding data points. Error bars represent the standard errors of the correlation coefficients.

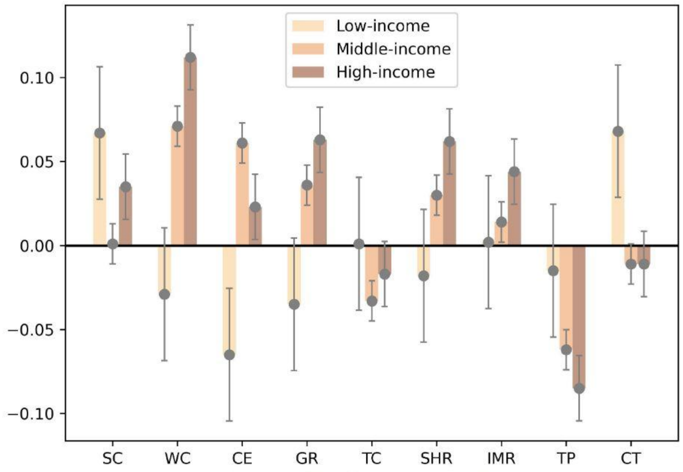

(a) Controling for income status

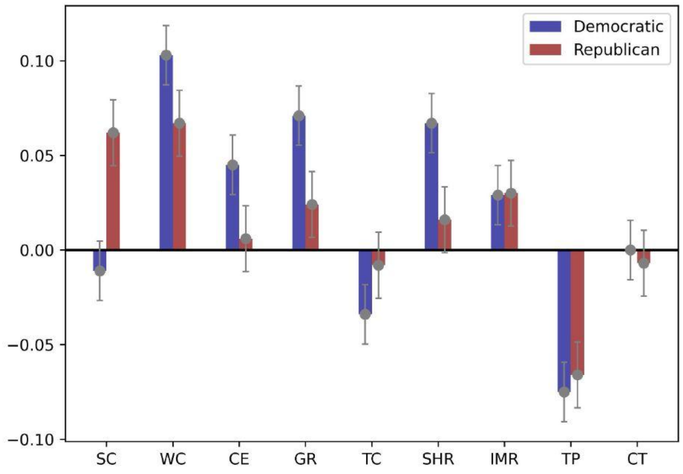

(b) Controling for party affiliation

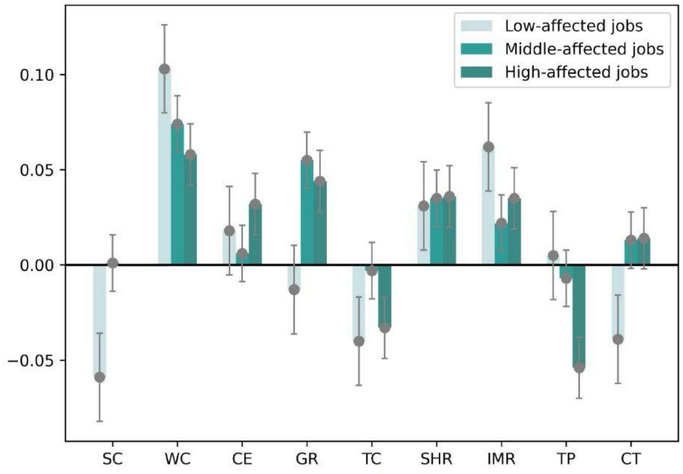

(c) Controling for job area

S11 Figure: The partial correlation between SWB-ladder variable and COVID-19 policy measures while controlling for respondents' specific job areas.

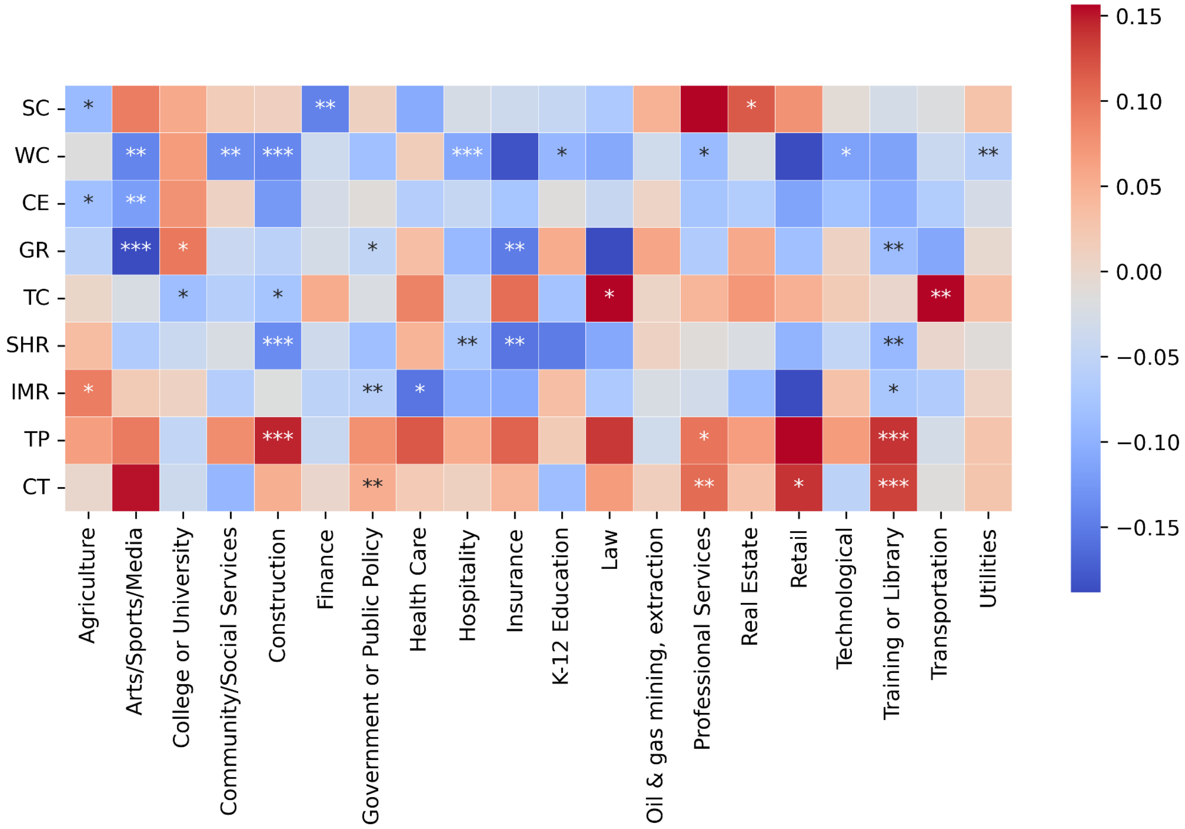

S12 Figure: The partial correlation between Enjoyment variable and COVID-19 policy measures while controlling for respondents' specific job areas.

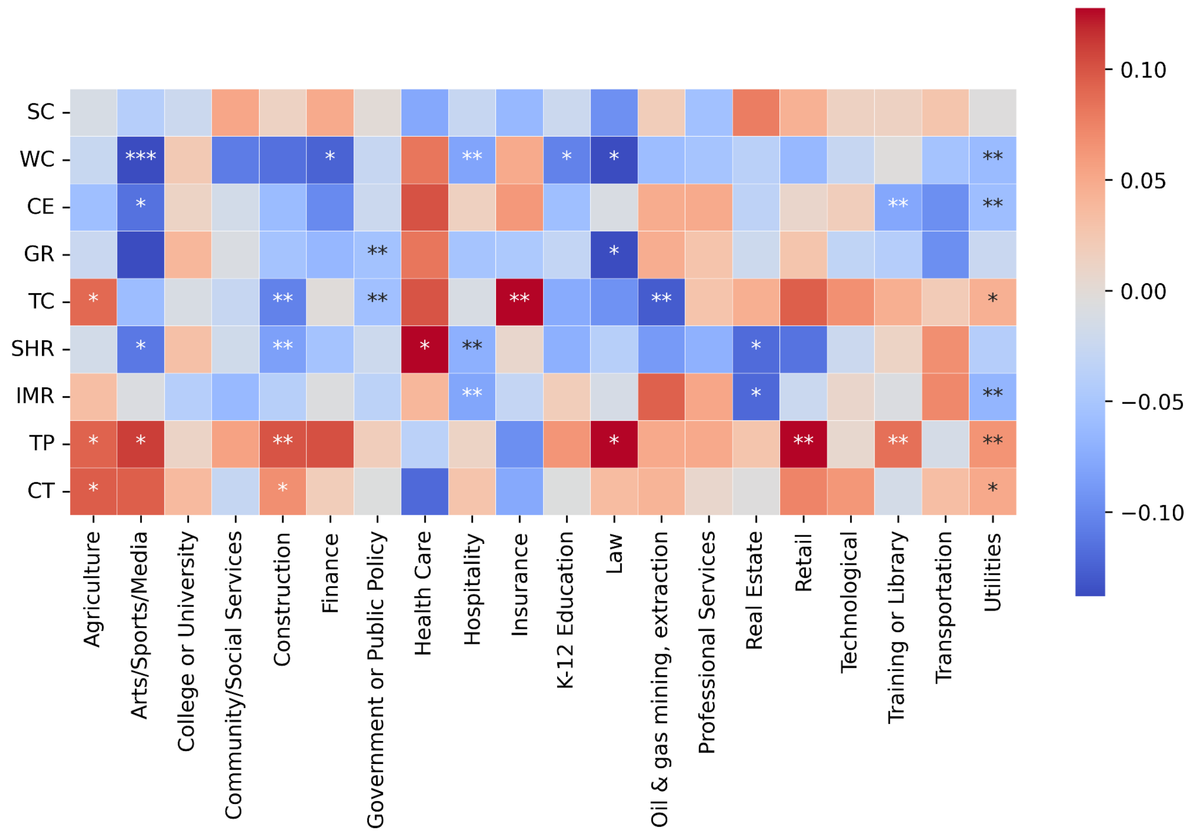

S13 Figure: The partial correlation between Worry variable and COVID-19 policy measures while controlling for respondents' specific job areas.

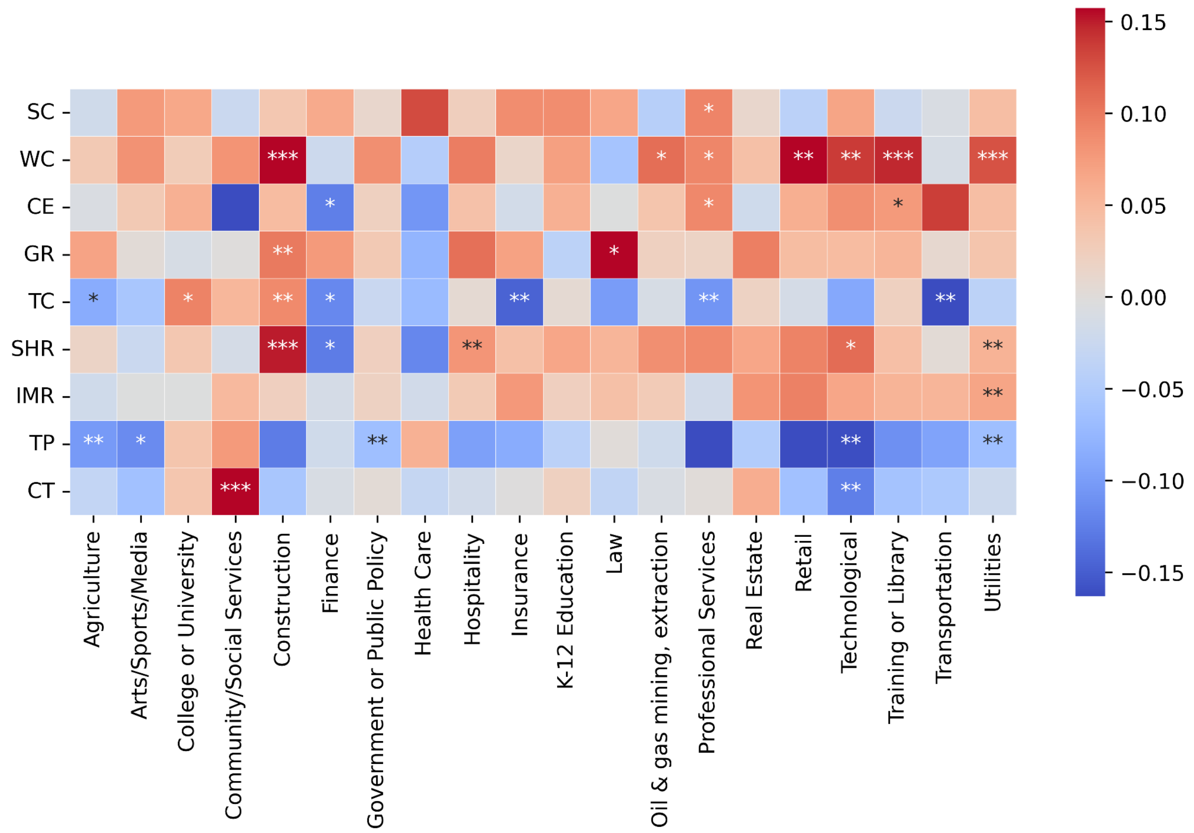

S14 Table: Fixed-effects regression results of the internal movement restrictions (IMR), testing policy (TP), and contact tracing (CT) policies.

			**Internal movement restrictions**	**Testing policy**	**Contact tracing**
Dummy Varibles	policy (Compared to level 0 (no measures))	Level 2	-0.034	-0.072	0.084
		Level 3	-	-0.084	-
	Gender (Compared to female)	Male	0.210 ***	0.210 ***	0.210 ***
	Employed Situation (Compared to full-time)	Part-time	-0.083	-0.084	-0.084
	Job Area (Compared to Agriculture, Forestry, Fishing, and Hunting)	Middle-affected	0.156 **	0.158 **	0.156 **
		High-affected	0.094	0.096	0.094
	HasChildUnder12 (Compared to No)	Yes	-0.173 **	-0.175 **	-0.172 **
	HasChild12-17 (Compared to No)	Yes	-0.186 **	-0.187 **	-0.186 **
	Income (Compared to low income)	Middle income	0.561 ***	0.560 ***	0.559 ***
		High income	0.885 ***	0.882 ***	0.884 ***
	Party (Compared to Demographic)	Republican	0.285	0.284	0.286
	state (Compared to AK)	NH	-0.199	-0.207	-0.188
		MI	1.258	1.250	1.323
Numeric Variables	Age		0.010 ***	0.010 ***	0.010 ***
	No. Children		0.056 **	0.056 **	0.056 **

	Confirmed	0.181	0.197	0.182
	Deaths	-0.287	-0.299	-0.286
